# FARMS: A New Algorithm for Variable Selection

**DOI:** 10.1155/2015/319797

**Published:** 2015-07-26

**Authors:** Susana Perez-Alvarez, Guadalupe Gómez, Christian Brander

**Affiliations:** ^1^AIDS Research Institute IrsiCaixa-HIVACAT, Hospital Universitari Germans Trias i Pujol, Universitat Autònoma de Barcelona, 08916 Badalona, Spain; ^2^Universitat Politècnica de Catalunya, 08034 Barcelona, Spain; ^3^Institució Catalana de Recerca Avançada (ICREA), 08010 Barcelona, Spain; ^4^University of Vic and Central Catalonia (UVIC-UCC), 08500 Vic, Spain

## Abstract

Large datasets including an extensive number of covariates are generated these days in many different situations, for instance, in detailed genetic studies of outbreed human populations or in complex analyses of immune responses to different infections. Aiming at informing clinical interventions or vaccine design, methods for variable selection identifying those variables with the optimal prediction performance for a specific outcome are crucial. However, testing for all potential subsets of variables is not feasible and alternatives to existing methods are needed. Here, we describe a new method to handle such complex datasets, referred to as FARMS, that combines forward and all subsets regression for model selection. We apply FARMS to a host genetic and immunological dataset of over 800 individuals from Lima (Peru) and Durban (South Africa) who were HIV infected and tested for antiviral immune responses. This dataset includes more than 500 explanatory variables: around 400 variables with information on HIV immune reactivity and around 100 individual genetic characteristics. We have implemented FARMS in *R* statistical language and we showed that FARMS is fast and outcompetes other comparable commonly used approaches, thus providing a new tool for the thorough analysis of complex datasets without the need for massive computational infrastructure.

## 1. Introduction

An important goal of biosciences research is the identification of traits, markers, or features associated with an outcome of interest. Such analyses may include complex datasets that can include thousands of host and pathogen genetic markers as well as clinical and experimental parameters that need to be probed for possible associations. One such challenging situation is the infection with Human Immunodeficiency Virus (HIV). In this case, the design of an effective HIV vaccine depends on a comprehensive delineation of immune correlates of controlled infection that factors in host genetic determinants, clinical parameters, and viral replication data. Regression models, which interrogate possible mathematical relation(s) between the outcome of interest and a subset of relevant predictive characteristics, provide a convenient framework for this identification [1, 2]. In this paper, we have combined biostatistics and bioinformatics knowledge to present a new regression method, FARMS, and demonstrate its utility in an exemplary study where the relationship between immune responses to HIV and relative disease control is being explored.

A crucial and difficult step when building a regression model is the selection of variables to be included in the final model from a set of plausible and meaningful variables [3]. The selection problem has been widely discussed: Hocking noted the importance and interest of selecting the best subset of variables and concluded that there is no unique answer to this problem [4]. Indeed, variable selection can be seen as a special case of the model selection problem where each model under consideration could correspond to a distinct subset of explanatory variables [5–7]. There is a rich literature discussing the appropriateness of stepwise methods, computational efficiency aspects, or False Discovery Rate selection control [8–13].

When trying to define the best subset of explanatory variables, one would be tempted to perform an all subsets regression analysis in order to identify the best predictive subset given a certain optimization criterion. However, if the number *n* of variables in the original dataset is exceedingly large, an exhaustive test of all possible variable subsets is generally not feasible, since fitting all the possible models can be prohibitively time-intensive [14, 15]. Recognizing these limitations, several methods have been proposed to reduce the model space, although this reduction might lead to a lack of interpretability and usability [7]. Some authors have proposed rules for shrinkage methods, suggesting that one could safely evaluate problems when there are at most 10 to 15 times more variables than observations, while others have established criteria to delete variables, for example,* Least Absolute Shrinkage and Selection Operator* (LASSO) [11, 16–18].

In this paper, we present FARMS, as acronym for forward and all subsets regression for model selection, a new algorithm for model selection based on Liebminger et al.'s proposal [19]. The unique feature of this algorithm is that it combines forward variable selection and all subsets regression with improved prediction performance. Importantly, it also requires less computational time than comparable approaches. The motivation to develop this algorithm was the urgent need to define immune parameters of controlled HIV infection that could guide HIV vaccine development. The special challenges with such analyses are the extreme outbred population structure of the human host organism, the extensive number of T cell responses an individual can mount in response to HIV infection, and viral parameters, especially viral genome sequence polymorphisms.

HIV disease status is commonly assessed by determining an individual's CD4 T cell count and his/her steady state viral load, also referred to as viral set point. It is this viral set point that largely predicts how fast an individual will develop AIDS. However, the mechanisms that determine the in vivo viral set point remain poorly defined and likely include a multitude of factors, including viral replicative fitness, viral sequence diversity and host genetics as well as quantitative and qualitative determinants of the cellular and humoral host immune response [20]. The cellular immune response to HIV, mediated by CD4+ and CD8+ T cells, can be assessed by relatively straight forward in vitro analyses that determine the number of T cells in the body that can interact with short antigenic viral determinants, referred to as “T cell epitopes.” To this end, an in vitro ELISPOT assay can be employed in which an individual's peripheral blood T cells are incubated with synthetic peptides (generally 15–20 amino acids in length) representing the viral proteome [21, 22]. In our analyses, there are 410 such partially overlapping peptides (OLP), numbered from 1 to 425, as there are some gaps in the not entirely continuous numbering [21]. Of importance, reactivity to these 410 OLP depends, among other factors, on the presence or absence of genes in the human leukocyte antigen (HLA) [23] locus on chromosome 6, particularly the HLA class I alleles [24]. These HLA class I alleles encode specialized molecules that present small viral peptides on the surface of infected cells to host's CD8 T cells, also referred to as cytotoxic T lymphocytes (CTL). The classical HLA class I alleles include HLA-A, HLA-B, and HLA-C alleles, of which each individual encodes 2 versions that are inherited from the parents. There are close to 10,000 different allelic variants for the HLA-A, HLA-B, and HLA-C loci together (http://hla.alleles.org/nomenclature/stats.html). For each CD8+ T cell immune response detected in in vitro assays one of the 6 HLA class I molecules “restricts” a T cell response; that is, the T cell can react with viral antigen in the context of this class I molecule. For effective antiviral immunity and vaccine design alike, it is thought important that many HLA class I alleles can be involved in the immune defense both on the individual level and the population level as this ensures a broad antiviral T cell reactivity. As a consequence, large population screenings in HIV-infected individuals will provide a multitude of genetic, clinical, and experimental parameters. When intending to define best models of OLP reactivity and host HLA genetics that are associated with relative HIV control, this considerable number of parameters poses a formidable challenge for the necessary in-depth analysis.

## 2. Material and Methods

### 2.1. Datasets

The datasets included patients infected with HIV from two geographic areas: 631 patients from Durban (South Africa) and 236 patients from Lima (Peru) form two different cohorts with population-specific host genetics and immune reactivity data [25]. These two datasets were analyzed by means of FARMS aiming at assessing the predictive value of immune and genetic parameters for in vivo HIV viral loads in these untreated subjects. Protocols were approved in Lima by the IMPACTA Human Research Committee and in Durban by the Ethical Committee of the Nelson R. Mandela School of Medicine at the University of KwaZulu-Natal. All subjects provided written informed consent. We gathered information about HIV viral load, human leukocyte antigen [23] polymorphisms, and the reactivity of each of 410 individual overlapping peptides (OLP) spanning the entire viral proteome. The main characteristics of this cohort are summarized in Table 1. Although the HIV viral load of the patient, given as number of viral copies per mL, is a continuous variable, we note that there exists a lower detection limit depending on the technology used. In the present case, the lower detection limit was 50 copies/mL and, for the purpose of the analyses, the values were set as 49 copies/mL. The Box-Cox transformation was used to normalize the data: the lambda parameter was estimated (lambda = 0.061) and log10 transformation was applied accordingly. After logarithmic transformation, the data normality was assessed graphically by histograms, density plot and* Q-Q* plot as well as by the Shapiro-Wilk test.

Information on the 2 HLA-A, 2 HLA-B, and the 2 HLA-C alleles that every individual possesses was collected for both cohorts. The HLA genes in the Peruvian cohort were determined using a method that was sufficiently sensitive to discriminate between different subtypes (i.e., variants) of alleles at a 4-digit precision, while the Durban data consisted of two-digit typing. New binary variables were generated, in both Lima and Durban datasets, as follows: (i) for each allelic variant on each of the three HLA loci (HLA-A, HLA-B, and HLA-C) a binary variable was created with value 1 if the subject had that respective variant and 0 otherwise; (ii) for each OLP a binary variable was set with value 1 if the subject has responded to this OLP and 0 otherwise.

### 2.2. FARMS Algorithm

FARMS is an algorithm that works by iterating several steps according to a specified criterion. Liebminger et al. proposed in 2007 [19] an algorithm for model selection that combined forward variable selection and all subsets regression. In their work, they compared their proposed algorithm with a genetic algorithm and the standard stepwise method applying all of them to three different datasets. They described how their algorithm selected models with improved prediction performance in addition to requiring less computational time. We have modified this algorithm in order to include additional features and lend it more flexibility according to our specific needs when analyzing HLA genetic data and human immune reactivity data against HIV. The FARMS algorithm is available on the GRASS web page at http://grass.upc.edu/software/farms_code/view.

**Figure 1:**
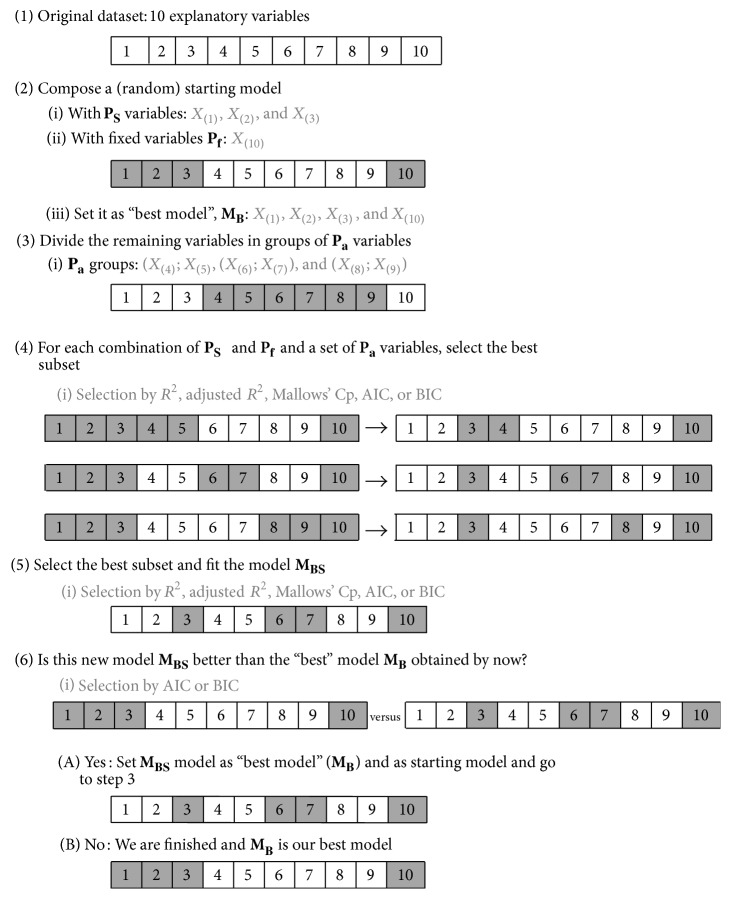
Illustration of FARMS algorithm. On a dataset of 10 covariates, variable “10” is forced to be always included. The starting model includes 3 variables (in addition to the forced-in ones) and adds 2 more variables in each iteration.

The basic FARMS algorithm is illustrated in Figure 1 by means of a toy example consisting of a dataset of 10 variables. Let *P* be the set of all potential explanatory variables of the outcome of interest. Define two subsets within *P*:

(a) *P*_*s*_: the set of variables included in an initial model. This set of variables is the starting point for the exploration of *P*.
(b) *P*_*F*_: the set of variables to be forced into a final model, not including those already in *P*_*s*_.

The algorithm iterates over the following steps:

(1) Start by building model *M*_*B*_ with the variables *P*_*s*_ and *P*_*F*_.
(2) The subset of variables not included in *M*_*B*_ is divided into user-defined fixed-sized groups of variables: *P*_*a*_.
(3) For each *P*_*a*_, build a new model based on fitting the variables *P*_*a*_, *P*_*s*_, and *P*_*F*_ and select the best subset of variables according to a predefined criterion *C*_1_.
(4) Select the best model *M*_*BS*_ among all the best subsets obtained in step (3) following the criterion *C*_1_.
(5) Compare models *M*_*B*_ and *M*_*BS*_. While *M*_*BS*_ is better than *M*_*B*_, define as *P*_*s*_ the explanatory variables included in *M*_*BS*_, go to step (2) and start all over or until a user-defined maximum time of computation is reached. Otherwise go to step (6).
(6) *M*_*B*_ is declared the best model and the algorithm finishes.

The user can choose to specify

(i) the criterion *C*_1_ to select a subset of variables among a group of them,
(ii) the criterion *C*_2_ to select between two candidate models,
(iii) the “starting” variables *P*_*S*_ in the initial model: the user can specify the size of the set *P*_*S*_ as well as the initial variables. If this is not specified, FARMS has a default size value and by default chooses *P*_*S*_ randomly from all the available variables, that is, within *P*-*P*_*F*_,
(iv) the variables forced into the final model *P*_*F*_: the user can specify some variables that should always be included in the final selected model or leave this option blank and allow for the selection of variables among all the ones included in the original dataset (*P*),
(v) the total number of variables into the final model can be fixed as well. By default, this number is the total number of variables in the dataset; so the best model could, a priori, include all the available variables.

The selection of the best model can be made according to any of the following criteria: Mallows' Cp [26], Akaike's information criterion (AIC) [27], and the Schwarz or Bayesian information criterion (BIC) [28]. The best subset selection can be additionally based on the *R*-square, the adjusted *R*-square, and the residual sum of squares (RSS). All these criteria originated from very different points of view: Mallows' Cp measures the performance of the variables in terms of the standardized total mean squared error of prediction (MSE) providing a balance between the lack of fit and the complexity of the model for the observed data. The AIC selects the candidate model that minimizes the estimated information lost when the model is used to approximate the process that generated the observed data (full reality) and it is a good option for prediction. The BIC, optimal for explaining the relationship between outcome and covariates, selects the model that maximizes the posterior probability applying a severe penalty term for the number of parameters in the model. Essentially, AIC and BIC will give similar answers with BIC leading to simpler models than AIC because it applies a larger penalty for complex models [29, 30]. Finally, the residual sum of squares, the coefficient of determination *R*-square, and the adjusted *R*-square are used [14] to measure the overall fit of a model, with the latter being the preferred alternative because it penalizes the *R*-square when extra variables are included in the model.

**Figure 2:**
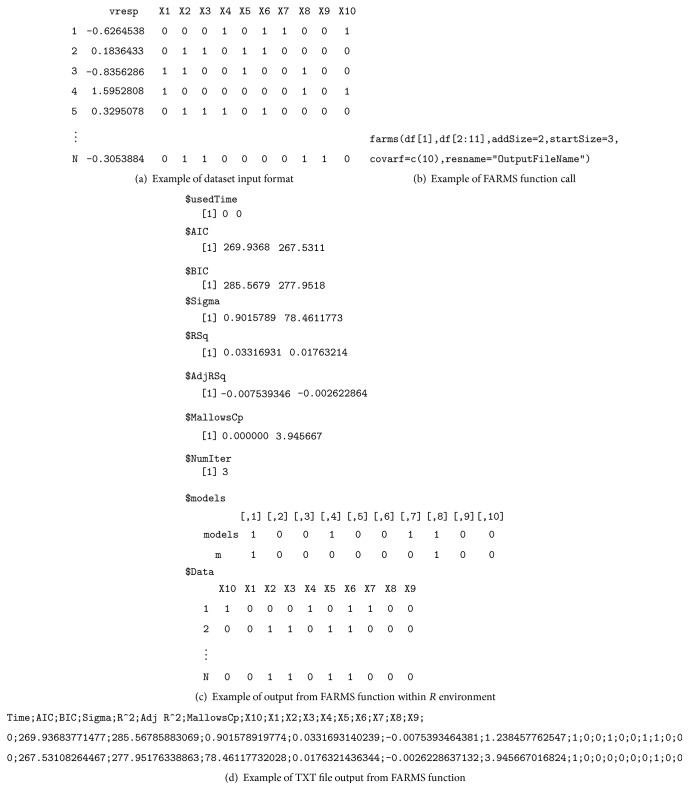
Illustration of FARMS input and output. (a) FARMS function requires data as *R* data frame containing all the variables (response and explanatory variables). (b) Calling the FARMS function within *R* requires the indication of at least the response variable and the explanatory variables. In this illustration, which follows the explanation of Figure 1, we also indicate the number of variables to add in each iteration, the number of variables to compose the starting model, the columns containing the forced-in variables, and the name of the output file. (c) By default, FARMS function returns an *R* object containing information for each iteration (two iterations in this illustration) and the dataset as processed by the algorithm. (d) Optionally, FARMS function can produce a text file containing the same information as the *R* object output, adding a text line for each iteration, helping also to monitor the algorithm execution and to track the evolution of models until the final model is obtained.

Data input and output formats to use FARMS are kept very user-friendly. In brief, for data input, any database in a format that *R* can read (i.e., an Excel, TXT, or CSV file) is suitable. Alternatively, data files generated by any statistical software that are compatible with *R* (such as SPSS or Stata) can be used. FARMS output is composed of 2 parts: first, an external txt file, written by *R* on the working directory, that adds a line on each algorithm iteration indicating the following information: the number of iteration, time since beginning of execution, value of the AIC and the BIC for the selected model at this point in the analysis, and for each variable on the dataset, an indication whether it is included in the model in that iteration (value = 1) or not included (value = 0) and second, an *R* object within the software environment including the information of the last model selected (information already included in the file). This object allows the user to easily fit the selected/best model within the *R* environment and continue the data analysis. An illustration of data input format and data output format is shown in Figure 2.

### 2.3. Univariate Data Analysis

Univariate analysis for the two cohorts (Lima and Durban) was performed over each HLA subtype present (Supplementary Table  1 in Supplementary Material available online at http://dx.doi.org/10.1155/2015/319797) and each reactive OLP (Supplementary Table  2) and related to HIV viral loads. The two cohorts were both analyzed separately and pooled into a single dataset. Specifically, for each HLA allele and for each OLP present in the database, we wondered whether people having the allele under analysis and/or responding to the OLP under analysis showed a significantly higher or lower viral load than the rest of the cohort. These questions were addressed by means of a bilateral Student's *t*-test to compare values of viral loads between the two groups defined as having or not having a specific HLA allele or making a response or not to an individual OLP. Results were reported as *P* values for a 95% confidence and corrected with *q* values setting the False Discovery Rate (FDR) to 10%.

### 2.4. Regression Methods

The following regression methods other than FARMS were applied to these two cohorts in order to find a relationship between the viral load and more than one HLA or OLP at a time: all subsets regression, forward selection regression, backward stepwise regression, and forward stepwise regression which is a combination of the previous two and which is later referred to as “Forward Stepwise”. These regression methods, all of them criticized for not being able to reflect model uncertainty accurately enough, were included as benchmarks for FARMS performance. For the implementation of the regression methods, few further technicalities were taken into account:

(i) We randomly sorted HLAs and OLPs variables before applying FARMS or any of the other approaches to avoid any possible relation between the order of the variables in the dataset and either the time of execution or the obtained model.
(ii) For this methodological comparison we previously evaluated FARMS not only to assess its robustness but also to choose the best value for the parameters Ps and Pa in terms of time and optimal statistical properties in the model. Therefore, the number of variables in the starting model (*Ps*) and the number of variables to be added in each step (*Pa*) were set according to the best results.

### 2.5. FARMS Robustness

In order to assess how robustly FARMS performs, that is, how stable the final model remains as the number of variables included initially or added in each iteration of the algorithm was changed, we executed the algorithm by varying the values of the number of variables in the starting model and by changing the number of variables to add on each step, that is, the size of groups to divide the rest of variables. We let both parameters vary between 2 and 21 and ran FARMS for each one of the 400 possible combinations in two different scenarios with completely different variables, HLA alleles and OLP. It is important to remember that, on each execution, the starting model or starting variables were selected randomly as we did not specify which ones to use. As a by-product, we have used those executions to study the computational time needed and the number of iterations internally performed.

## 3. Results

### 3.1. Predicting HIV Viral Loads in terms of Response Rates to OLP and Presence of HLA Alleles

When we applied FARMS to the specific HIV-derived datasets presented here, individual OLP and OLP combinations were identified, which have a high predictability of viral load in chronically HIV infected subjects. Our FARMS based analyses also showed that OLP reactivity (i.e., the specificity of the T cell response to HIV) had a greater predictability than host genetics (HLA class I genes) [25]. From a vaccine point of view, this revelation is crucial as it indicates that individuals with poor HLA genetics still have a possibility to mount effective T cell responses to HIV and that vaccine immunogen design could profit from a focus on such relevant regions in the viral proteome.

### 3.2. FARMS Evaluation

The best model selected in all the 400 FARMS runs was always the same regardless of the number of variables included initially or added in each iteration of the algorithm in either of the two different scenarios described at the end of Section 2. This homogeneity was only altered 11 times when using the OLP variables. In 389 runs, the final selected model contained a total of 12 variables with BIC = 2224.82. However for the remaining 11 combinations the final selected model included 15 variables, 5 of them different from the previous 12, and had a slightly smaller BIC (2218.85). In terms of prediction, the dominant model has a coefficient of determination close to 15% in contrast to 18% in the less frequent model.

**Figure 3:**
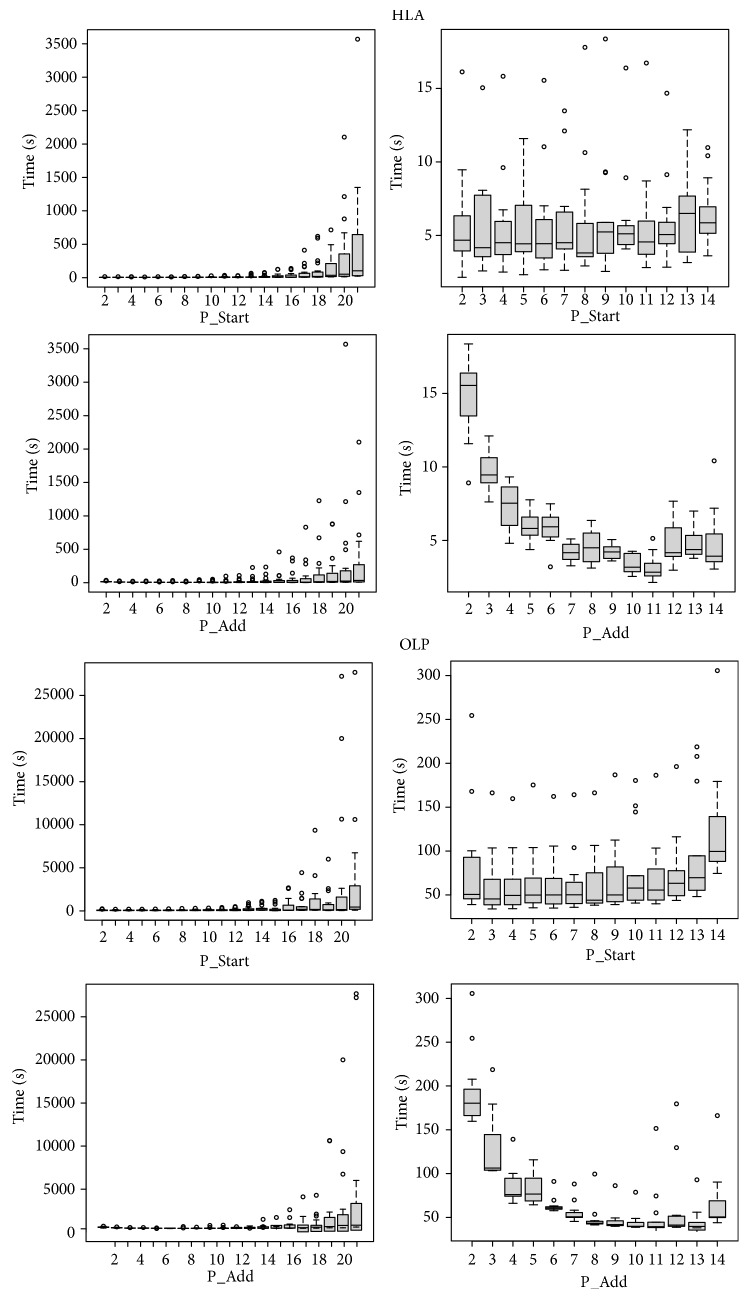
Evolution of the computing time (in seconds) needed to reach the final model according to FARMS parameters: starting number of covariates (P_Start) and number of covariates added in each step (P_Add). First two rows of figures refer to the HLA-only model; rows 3 and 4 correspond to OLP-only covariates model.

As expected, these two varying parameters affected the number of iterations that FARMS needed to reach the best model and also impacted the computational time required.  Figure 3 shows the evolution of time until the best model is selected for the HLA-only and the OLP-only models according to the number of variables in the starting model and the size of the adding subset. These results indicate that the number of covariates in the starting model does not have a strong effect on the total time of execution, although a large number of variables produce more variability: when the number of starting covariates is larger than 15, the time needed increases more than 50-fold. Compared to the starting size of variables, the number of variables to be added on each step has a much more profound effect on the total time of execution: as the number of variables to be added increases, the time also increases.

From these modulations and in light of the aims of the study (i.e., identifying a limited number of OLP that could be incorporated into an HIV vaccine sequence), we determined the optimum values of these two parameters: the number of variables for the starting model was set to 10, and the number of adding variables was set to 8.

### 3.3. FARMS Performance in Head-to-Head Comparisons with Other Methods

In order to compare the performance of FARMS with other established approaches, the data from Lima and Durban were used (Table 2). All subsets regression analysis exceeded one month of execution time for both scenarios (HLA and OLP variables), thus not providing us with a precise time to completion. The analysis using backward stepwise algorithm needed much more computational time, at least 3-fold longer, than the other stepwise approaches or FARMS and was thus only run once. The selected models using forward regression or forward stepwise took longer than FARMS when the selecting criterion was either the AIC or the BIC.

Models obtained with stepwise methods varied as we changed their parameters: selection by AIC or BIC and intercept-only baseline model or with forced-in variables. When setting the more appropriate parameters to approximate FARMS and stepwise methods (selection based on BIC and setting the model with forced-in variables as baseline model), forward selection provided a slightly worse model when using HLA variables; otherwise, the models obtained with FARMS and stepwise methods were the same and only differed in the time needed. These comparisons further confirm the validity of FARMS approach and demonstrate its favorable computational time.

**Figure 4:**
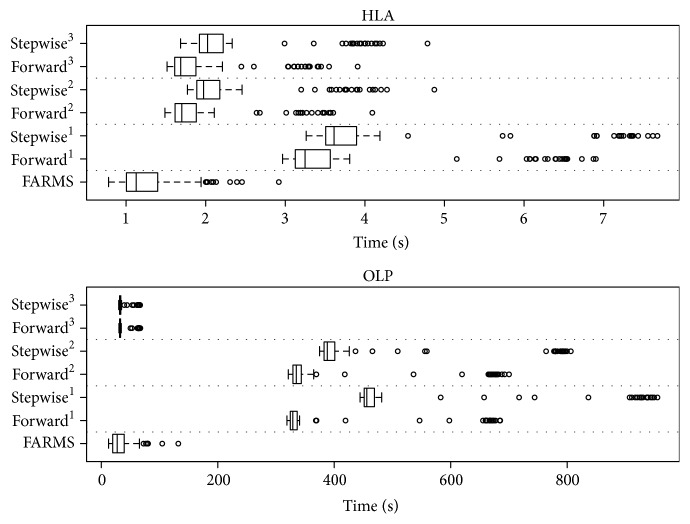
Comparison of computational time between FARMS, forward selection, and forward stepwise regression algorithms for the two possible scenarios, HLA-only and OLP-only. (1: selection by AIC and base model with intercept-only; 2: selection by AIC and base model with forced-in variables; 3: selection by BIC and base model with forced-in variables).

To further investigate the time differences between FARMS and more comparable stepwise approaches, we ran all the methods in the two scenarios (HLA and OLP) 100 times and compared their execution time by Student's *t*-test: Figure 4 summarizes the time of execution needed by each approach in addition to the time used in each run. FARMS was significantly faster than stepwise approaches applied to HLA-only covariates (76 covariates): FARMS usually needed one second, whereas both forward regression and forward stepwise approaches needed up to 3 times longer to completion (*F*-test *P* value < 2.2*e* − 16). This difference increased and showed FARMS to be also significantly faster when the OLP-only (406 covariates) dataset was evaluated as FARMS completed the task roughly 10x faster than standard forward or stepwise approaches (*F*-test *P* value < 2.2*e* − 16) (Table 1). Thus, FARMS offers a novel, versatile, and flexible approach that is not dependent on excessive computational power and runs in widely available *R* packages.

## 4. Discussion

We here report the development and performance of a new algorithm for variable selection referred to as FARMS. Overall, this new algorithm, when applied to high-dimensional datasets, yields better results in less time compared to other regression approaches (stepwise or all subsets techniques).

Stepwise algorithms use as a starting model either the intercept-only or the full model and build on this model by introducing or excluding variables one by one with no reference to other variables until the best model is selected according to an information criterion. The method we propose here starts with a random model containing some variables but not a full model and improves it by selecting subsets of variables with different sizes, allowing the algorithm to include in a single step more than one variable.

The common information criterion used in model selection is the AIC, but selection by BIC has been suggested to represent a better option for our specific situation: BIC penalizes the number of covariates, helping our goal of finding best model with fewer covariates. In addition, the BIC criterion leads to the final model faster when the dataset contains a large number of covariates. FARMS also implements other criteria that facilitate obtaining better models according to their prediction capacity, such as *R*-square or adjusted *R*-square. These improvements in the FARMS implementation allow for making a better comparison of those methodologies and enable the user to change the model selection according to the purpose of the data analysis.

Additional features that can be readily implemented in FARMS and can be very useful for statistical analyses include the evaluation of quadratic terms (the square of the value of each covariate), which can help to explain nonlinear relationship among the variables. In addition, FARMS offers the possibility of introducing the evaluation of the interaction terms, although for large datasets this may increase completion time considerably (but still less than needed by common approaches such as stepwise). Finally, the FARMS strategy can also be extended to other regression approaches, such as logistic regression, which is widely used in the biomedical and genetics field, as there are already packages in *R* that select best subsets of variables for logistic regression.

## Supplementary Material

Supplementary Table 1 outlines the composition of the rat milk replacer which was developed to closely represent maternal rat milk in composition (Wombaroo Food Products SA, Australia). The formula provides artificially reared pups with nutrients (energy, lipids and protein) for growth.

## Figures and Tables

**Table 1:** Data description. Summary of cohort characteristics either joined or split by country (Lima and Durban) showing viral load and its transformation log10 distribution. Below, number of HLA alleles and OLP present in the database and frequency of the top 5 parameters for each category.

	Viral load			Log10 (Viral load)		
	Global	Lima	Durban	Global	Lima	Durban
Median	37800	37240	37900	4.577	4.571	4.579
IQR	(8715; 131500)	(13310; 109000)	(7075; 138500)	(3.94; 5.119)	(4.124; 5.038)	(3.85; 5.141)

	HLA			OLP		
	Global	Lima	Durban	Global	Lima	Durban

Total number	73	62	66	406	391	371
1st	C∗07	A∗02	B∗15	76	76	78
	(*N* = 310; 6.31%)	(*N* = 168; 12.80%)	(*N* = 233; 6.48%)	(*N* = 293; 2.77%)	(*N* = 76; 2.09%)	(*N* = 234; 3.37%)
2nd	A∗02	B∗35	C∗07	78	84	76
	(*N* = 294; 5.99%)	(*N* = 99; 7.54%)	(*N* = 222; 6.17%)	(*N* = 284; 2.69%)	(*N* = 63; 1.74%)	(*N* = 217; 3.12%)
3rd	B∗15	C∗04	A∗30	84	81	84
	(*N* = 278; 5.66%)	(*N* = 88; 6.70%)	(*N* = 205; 5.70%)	(*N* = 262; 2.48%)	(*N* = 61; 1.68%)	(*N* = 199; 2.86%)
4th	A∗30	C∗07	C∗06	25	85	25
	(*N* = 225; 4.58%)	(*N* = 88; 6.70%)	(*N* = 194; 5.39%)	(*N* = 192; 1.82%)	(*N* = 53; 1.46%)	(*N* = 178; 2.56%)
5th	C∗04	B∗39	A∗68	41	78	41
	(*N* = 217; 4.42%)	(*N* = 59; 4.49%)	(*N* = 162; 4.50%)	(*N* = 190; 1.80%)	(*N* = 50; 1.38%)	(*N* = 151; 2.17%)

**Table 2:** Comparison of results when basing variable selection on the FARMS algorithm or common strategies. The number of covariates included in the final model excludes the “forced in” covariates. FARMS parameters used in this case are number of adding covariates = 8, number of starting covariates = 10, and the selecting criteria for both best subset and best model was the BIC. (All runs were executed on an Intel Xeon x5680 machine with 6 CPU cores and 95 GB RAM memory under a Linux Suse 11.0 OS).

		Time (seconds)^*^ Mean (IQR)	Number of vars.^**^	BIC	AIC	*R* ^2^	Adj. *R* ^2^
HLA	FARMS	**1.27 (1.00; 1.40)**	**9**	**2235.4**	**2183.03**	**11.67%**	**10.74%**
	All subsets	>1 month	—	—	—	—	—
	Forward selection^1^	3.84 (3.13; 3.52)	17	2259.4	2168.86	14.69%	12.98%
	Forward stepwise^1^	4.32 (3.51; 3.90)	17	2259.4	2168.86	14.69%	12.98%
	Forward selection^2^	2.01 (1.62; 1.88)	10	2235.45	2178.27	12.36%	11.33%
	Forward stepwise^2^	2.35 (1.89; 2.18)	9	2235.44	2183.03	11.67%	10.74%
	Forward selection^3^	1.99 (1.61; 1.88)	10	2235.45	2178.27	12.36%	11.33%
	Forward stepwise^3^	2.38 (1.92; 2.22)	9	2235.44	2183.03	11.67%	10.74%
	Backward stepwise^3^	13 s	10	2236.52	2174.58	12.93%	11.81%

OLP	FARMS	**33.4** **(19.47; 37.95)**	**12**	**2224.8**	**2158.11**	**14.77%**	**13.57%**
	All subsets	>1 month	—	—	—	—	—
	Forward selection^1^	393.2 (324.40; 336.60)	79	2396.53	2010.56	38.40%	32.22%
	Forward stepwise^1^	545.9 (451.70; 469.70)	83	2415.46	2010.53	38.97%	32.51%
	Forward selection^2^	401.7 (329.50; 343.40)	80	2403.3	2012.56	38.40%	32.13%
	Forward stepwise^2^	462.4 (382.50; 401.50)	76	2385.18	2013.51	37.76%	31.77%
	Forward selection^3^	38.09 (31.34; 33.11)	12	2224.82	2158.11	14.77%	13.57%
	Forward stepwise^3^	38.31 (31.34; 33.43)	12	2224.82	2158.11	14.77%	13.57%
	Backward stepwise^3^	>12 hours	23	2232.63	2108.63	21.68%	19.45%

^1^Selection by AIC and base model with intercept-only.

^2^Selection by AIC and base model with forced-in variables.

^3^Selection by BIC and base model with forced-in variables.

^*^Time obtained after 100 executions for each scenario.

^**^Including forced-in variables.
