# Supplementary material for: FARMS: A New Algorithm for Variable Selection
Source: Biomed Res Int. 2015 Jul 26;2015:319797. doi: 10.1155/2015/319797 (PMC4529908; doi:10.1155/2015/319797)
Supplement: Supplementary file 1 — Supplementary Table 1 outlines the composition of the rat milk replacer which was developed to closely represent maternal rat milk in composition (Wombaroo Food Products SA, Australia). The formula provides artificially reared pups with nutrients (energy, lipids and protein) for growth. [file 319797.f1.pdf]

Supplementary Tables

ST1. Univariate analysis results for HLAs. Highlighted those results with p-value<=0.1 or q-value<=0.1.

	Global						Durban Cohort						Peruvian Cohort					
	N		Mean (Std.Dev) Log10 VL		P-value	Q-value	N		Mean (Std.Dev) Log10 VL		P-value	Q-value	N		Mean (Std.Dev) Log10 VL		P-value	Q-value
	HLA-	HLA+	HLA-	HLA+			HLA-	HLA+	HLA-	HLA+			HLA-	HLA+	HLA-	HLA+		
**HLA.A.01**	797	70	4,49 (0,88)	4,41 (1,05)	0,5287	0,7961	574	57	4,48 (0,93)	4,30 (1,10)	0,2396	0,4296	223	13	4,52 (0,73)	4,89 (0,64)	0,0694	0,2461
**HLA.A.11**	852	15	4,49 (0,90)	4,15 (0,62)	0,0528	0,2951	628	3	4,47 (0,95)	4,25 (0,37)	0,4084	0,5618	224	12	4,57 (0,73)	4,12 (0,68)	0,0499	0,2274
**HLA.A.31**	840	27	4,48 (0,90)	4,70 (0,74)	0,1379	0,5094	627	4	4,46 (0,95)	5,39 (0,24)	0,0025	0,0148	213	23	4,54 (0,73)	4,58 (0,74)	0,7767	0,5162
**HLA.A.68**	669	198	4,45 (0,89)	4,60 (0,90)	0,0365	0,2353	469	162	4,41 (0,95)	4,63 (0,92)	0,0094	0,0412	200	36	4,55 (0,72)	4,49 (0,79)	0,6434	0,4656
**HLA.A.74**	796	71	4,52 (0,87)	4,10 (1,08)	0,0019	0,0211	562	69	4,51 (0,92)	4,08 (1,09)	0,0023	0,0148	234	2	4,54 (0,73)	4,58 (1,12)	0,9724	0,5598
**HLA.B.08**	783	84	4,48 (0,90)	4,60 (0,85)	0,2198	0,5664	554	77	4,46 (0,96)	4,54 (0,86)	0,4038	0,5618	229	7	4,52 (0,73)	5,16 (0,25)	0,0001	0,0022
**HLA.B.13**	837	30	4,50 (0,89)	4,21 (0,97)	0,1201	0,5094	604	27	4,48 (0,95)	4,13 (0,97)	0,0767	0,2458	233	3	4,54 (0,73)	4,93 (0,63)	0,3888	0,4656
**HLA.B.15**	589	278	4,44 (0,90)	4,58 (0,89)	0,0386	0,2353	398	233	4,37 (0,95)	4,63 (0,93)	0,0010	0,0098	191	45	4,59 (0,75)	4,33 (0,62)	0,0146	0,1162
**HLA.B.18**	818	49	4,47 (0,90)	4,83 (0,69)	0,0008	0,0115	597	34	4,44 (0,95)	4,95 (0,72)	0,0003	0,0050	221	15	4,54 (0,74)	4,57 (0,56)	0,8750	0,5267
**HLA.B.35**	741	126	4,45 (0,92)	4,72 (0,72)	0,0003	0,0061	604	27	4,46 (0,95)	4,61 (0,91)	0,3915	0,5618	137	99	4,40 (0,75)	4,74 (0,66)	0,0002	0,0023
**HLA.B.39**	777	90	4,50 (0,90)	4,37 (0,83)	0,1498	0,5094	600	31	4,49 (0,94)	4,10 (1,02)	0,0480	0,1921	177	59	4,56 (0,74)	4,50 (0,69)	0,6285	0,4656
**HLA.B.42**	724	143	4,51 (0,90)	4,38 (0,87)	0,1257	0,5094	492	139	4,49 (0,97)	4,39 (0,89)	0,2524	0,4331	232	4	4,55 (0,73)	4,22 (0,18)	0,0241	0,1282
**HLA.B.49**	849	18	4,48 (0,89)	4,63 (1,10)	0,5828	0,8308	622	9	4,46 (0,95)	5,14 (0,95)	0,0638	0,2358	227	9	4,56 (0,71)	4,12 (1,03)	0,2388	0,4481
**HLA.B.57**	820	47	4,52 (0,88)	3,88 (0,93)	0,0000	0,0019	592	39	4,51 (0,94)	3,85 (0,95)	0,0001	0,0036	228	8	4,56 (0,72)	4,02 (0,90)	0,1376	0,3658
**HLA.B.81**	796	71	4,52 (0,87)	4,08 (1,05)	0,0009	0,0115	561	70	4,51 (0,93)	4,09 (1,05)	0,0017	0,0139	235	1	4,55 (0,73)	3,57 (0)		
**HLA.C.04**	650	217	4,48 (0,91)	4,52 (0,84)	0,5137	0,7961	502	129	4,50 (0,96)	4,35 (0,92)	0,1179	0,3148	148	88	4,41 (0,75)	4,77 (0,63)	0,0001	0,0022
**HLA.C.05**	842	25	4,48 (0,90)	4,75 (0,90)	0,1431	0,5094	622	9	4,46 (0,94)	5,21 (1,07)	0,0693	0,2378	220	16	4,55 (0,73)	4,50 (0,70)	0,8033	0,5172
**HLA.C.06**	658	209	4,43 (0,88)	4,65 (0,93)	0,0026	0,0246	437	194	4,38 (0,94)	4,65 (0,95)	0,0010	0,0098	221	15	4,53 (0,73)	4,68 (0,67)	0,4431	0,4656
**HLA.C.12**	823	44	4,51 (0,89)	4,10 (0,93)	0,0065	0,0542	602	29	4,49 (0,94)	3,92 (1,00)	0,0052	0,0251	221	15	4,55 (0,73)	4,44 (0,70)	0,5537	0,4656
**HLA.C.17**	711	156	4,50 (0,90)	4,43 (0,89)	0,3991	0,7484	479	152	4,48 (0,97)	4,44 (0,90)	0,6628	0,6924	232	4	4,55 (0,73)	4,22 (0,18)	0,0241	0,1282

ST2. Univariate analysis results for OLPs. Highlighted those results with p-value<=0.1 or q-value<=0.1.

	Global						Durban Cohort						Peruvian Cohort					
	N		Mean (Std.Dev) Log10 VL				N		Mean (Std.Dev) Log10 VL				N		Mean (Std.Dev) Log10 VL			
	HLA-	HLA+	HLA-	HLA+	P-value	Q-value	HLA-	HLA+	HLA-	HLA+	P-value	Q-value	HLA-	HLA+	HLA-	HLA+	P-value	Q-value
**OLP.3**	736	131	4,54 (0,89)	4,20 (0,88)	0,0001	0,0049	528	103	4,52 (0,94)	4,17 (0,94)	0,0006	0,0168	208	28	4,57 (0,74)	4,32 (0,60)	0,0510	0,5196
**OLP.6**	797	70	4,53 (0,89)	3,98 (0,79)	0,0000	0,0001	588	43	4,51 (0,94)	3,87 (0,86)	0,0000	0,0011	209	27	4,59 (0,73)	4,14 (0,64)	0,0017	0,0720
**OLP.7**	797	70	4,53 (0,88)	3,94 (0,95)	0,0000	0,0004	579	52	4,52 (0,93)	3,83 (0,99)	0,0000	0,0006	218	18	4,57 (0,72)	4,26 (0,79)	0,1265	0,6306
**OLP.10**	838	29	4,49 (0,90)	4,28 (0,75)	0,1375	0,3105	612	19	4,47 (0,96)	4,37 (0,74)	0,5713	0,5553	226	10	4,56 (0,72)	4,10 (0,78)	0,0978	0,6046
**OLP.11**	730	137	4,52 (0,90)	4,33 (0,85)	0,0208	0,1326	536	95	4,49 (0,96)	4,33 (0,91)	0,1179	0,3060	194	42	4,59 (0,73)	4,33 (0,71)	0,0379	0,5196
**OLP.12**	817	50	4,50 (0,90)	4,28 (0,78)	0,0587	0,2260	605	26	4,47 (0,96)	4,28 (0,75)	0,2151	0,3834	212	24	4,57 (0,71)	4,28 (0,82)	0,1020	0,6068
**OLP.15**	864	3	4,49 (0,90)	4,89 (0,23)	0,0896	0,2603	628	3	4,46 (0,95)	4,89 (0,23)	0,0780	0,2500	236	0	4,54 (0,73)	0 (0)	NA	NA
**OLP.18**	849	18	4,50 (0,89)	4,09 (0,87)	0,0653	0,2316	620	11	4,48 (0,95)	3,92 (0,90)	0,0700	0,2383	229	7	4,55 (0,73)	4,34 (0,83)	0,5406	0,9047
**OLP.19**	816	51	4,50 (0,89)	4,24 (0,95)	0,0580	0,2260	603	28	4,48 (0,95)	4,17 (0,97)	0,1143	0,3030	213	23	4,57 (0,70)	4,31 (0,94)	0,2233	0,7487
**OLP.21**	786	81	4,50 (0,90)	4,33 (0,88)	0,0925	0,2603	558	73	4,48 (0,96)	4,34 (0,90)	0,2100	0,3814	228	8	4,55 (0,73)	4,23 (0,65)	0,2070	0,7487
**OLP.22**	815	52	4,52 (0,89)	3,96 (0,90)	0,0001	0,0049	590	41	4,51 (0,94)	3,82 (0,92)	0,0000	0,0011	225	11	4,54 (0,74)	4,51 (0,63)	0,8462	0,9459
**OLP.23**	813	54	4,51 (0,89)	4,16 (0,86)	0,0054	0,0631	592	39	4,49 (0,95)	4,12 (0,95)	0,0246	0,1341	221	15	4,56 (0,74)	4,26 (0,55)	0,0607	0,5196
**OLP.24**	786	81	4,51 (0,90)	4,31 (0,89)	0,0599	0,2260	554	77	4,49 (0,96)	4,31 (0,88)	0,1127	0,3030	232	4	4,55 (0,72)	4,20 (1,11)	0,5768	0,9047
**OLP.25**	675	192	4,55 (0,88)	4,27 (0,94)	0,0003	0,0114	453	178	4,55 (0,94)	4,26 (0,94)	0,0008	0,0194	222	14	4,55 (0,72)	4,36 (0,85)	0,4235	0,9008
**OLP.26**	834	33	4,50 (0,89)	4,07 (0,92)	0,0113	0,1004	617	14	4,48 (0,95)	3,79 (0,85)	0,0096	0,0737	217	19	4,57 (0,71)	4,27 (0,94)	0,1964	0,7487
**OLP.27**	812	55	4,51 (0,90)	4,21 (0,78)	0,0085	0,0807	603	28	4,49 (0,95)	3,93 (0,84)	0,0018	0,0326	209	27	4,55 (0,74)	4,49 (0,61)	0,6488	0,9047
**OLP.29**	789	78	4,51 (0,90)	4,25 (0,87)	0,0149	0,1106	575	56	4,50 (0,95)	4,13 (0,87)	0,0039	0,0475	214	22	4,54 (0,72)	4,56 (0,84)	0,9413	0,9508
**OLP.30**	815	52	4,50 (0,90)	4,29 (0,83)	0,0912	0,2603	590	41	4,48 (0,95)	4,20 (0,88)	0,0531	0,2031	225	11	4,54 (0,74)	4,65 (0,48)	0,4945	0,9047
**OLP.31**	854	13	4,50 (0,89)	3,70 (0,78)	0,0031	0,0509	622	9	4,48 (0,95)	3,75 (0,75)	0,0190	0,1151	232	4	4,56 (0,72)	3,58 (0,97)	0,1373	0,6705
**OLP.33**	791	76	4,52 (0,88)	4,11 (1,00)	0,0008	0,0259	563	68	4,51 (0,93)	4,10 (1,05)	0,0026	0,0397	228	8	4,55 (0,74)	4,24 (0,41)	0,0714	0,5679
**OLP.34**	811	56	4,51 (0,88)	4,11 (1,00)	0,0043	0,0628	584	47	4,49 (0,94)	4,16 (1,05)	0,0417	0,1749	227	9	4,57 (0,72)	3,81 (0,70)	0,0117	0,2758
**OLP.37**	773	94	4,53 (0,89)	4,14 (0,90)	0,0001	0,0059	556	75	4,52 (0,94)	4,10 (0,95)	0,0006	0,0168	217	19	4,57 (0,73)	4,28 (0,66)	0,0928	0,6046
**OLP.39**	856	11	4,49 (0,90)	4,24 (0,89)	0,3801	0,4578	626	5	4,47 (0,95)	3,52 (0,59)	0,0218	0,1286	230	6	4,54 (0,73)	4,84 (0,61)	0,2756	0,8203
**OLP.41**	677	190	4,54 (0,89)	4,30 (0,90)	0,0015	0,0340	480	151	4,53 (0,95)	4,26 (0,93)	0,0023	0,0393	197	39	4,56 (0,73)	4,46 (0,73)	0,4465	0,9016
**OLP.42**	808	59	4,50 (0,90)	4,28 (0,76)	0,0366	0,1895	601	30	4,49 (0,95)	4,08 (0,77)	0,0089	0,0726	207	29	4,55 (0,73)	4,49 (0,70)	0,6593	0,9061
**OLP.43**	830	37	4,50 (0,90)	4,25 (0,83)	0,0874	0,2603	608	23	4,48 (0,95)	4,13 (0,92)	0,0904	0,2649	222	14	4,55 (0,74)	4,45 (0,61)	0,5846	0,9047
**OLP.45**	835	32	4,49 (0,90)	4,54 (0,79)	0,7184	0,5624	606	25	4,47 (0,95)	4,48 (0,88)	0,9171	0,6538	229	7	4,54 (0,74)	4,73 (0,21)	0,0656	0,5477
**OLP.46**	831	36	4,49 (0,90)	4,43 (0,82)	0,6592	0,5417	606	25	4,47 (0,95)	4,27 (0,91)	0,2785	0,4215	225	11	4,53 (0,74)	4,79 (0,40)	0,0678	0,5520
**OLP.48**	825	42	4,50 (0,90)	4,22 (0,86)	0,0471	0,2124	594	37	4,48 (0,95)	4,25 (0,90)	0,1338	0,3316	231	5	4,55 (0,73)	4,05 (0,48)	0,0757	0,5693
**OLP.51**	857	10	4,49 (0,90)	4,38 (0,64)	0,6021	0,5243	627	4	4,47 (0,95)	4,01 (0,35)	0,0741	0,2448	230	6	4,54 (0,73)	4,62 (0,70)	0,7889	0,9443
**OLP.55**	828	39	4,51 (0,89)	4,08 (0,99)	0,0130	0,1099	603	28	4,49 (0,94)	4,00 (1,00)	0,0175	0,1092	225	11	4,56 (0,71)	4,29 (1,00)	0,4087	0,8771
**OLP.56**	859	8	4,49 (0,90)	4,23 (0,46)	0,1606	0,3268	628	3	4,47 (0,95)	4,38 (0,69)	0,8560	0,6501	231	5	4,55 (0,73)	4,14 (0,33)	0,0450	0,5196
**OLP.59**	807	60	4,50 (0,90)	4,30 (0,79)	0,0630	0,2265	604	27	4,49 (0,95)	4,01 (0,86)	0,0090	0,0726	203	33	4,54 (0,74)	4,54 (0,65)	0,9510	0,9508
**OLP.60**	847	20	4,50 (0,89)	3,92 (0,80)	0,0049	0,0631	619	12	4,48 (0,95)	3,80 (0,89)	0,0243	0,1341	228	8	4,56 (0,73)	4,10 (0,67)	0,0966	0,6046
**OLP.63**	856	11	4,50 (0,89)	3,77 (0,69)	0,0061	0,0631	622	9	4,48 (0,95)	3,68 (0,74)	0,0120	0,0796	234	2	4,55 (0,73)	4,19 (0,00)	0,0000	0,0000
**OLP.159**	856	11	4,49 (0,89)	4,26 (1,18)	0,5342	0,5087	630	1	4,46 (0,95)	6,93 (0)	NA	NA	226	10	4,57 (0,72)	3,99 (0,82)	0,0554	0,5196
**OLP.160**	857	10	4,49 (0,89)	4,58 (1,31)	0,8351	0,5893	626	5	4,46 (0,95)	5,46 (0,96)	0,0789	0,2500	231	5	4,56 (0,72)	3,69 (0,98)	0,1160	0,6068

**OLP.163**	760	107	4,52 (0,91)	4,27 (0,78)	0,0030	0,0509	563	68	4,49 (0,96)	4,25 (0,85)	0,0296	0,1538	197	39	4,59 (0,74)	4,31 (0,62)	0,0174	0,3324
**OLP.165**	862	5	4,49 (0,90)	4,48 (0,78)	0,9895	0,6328	629	2	4,46 (0,95)	5,23 (0,19)	0,0882	0,2649	233	3	4,55 (0,73)	3,99 (0,52)	0,2006	0,7487
**OLP.167**	858	9	4,48 (0,90)	4,80 (0,71)	0,2195	0,3896	627	4	4,46 (0,95)	4,69 (1,07)	0,6987	0,5899	231	5	4,54 (0,73)	4,89 (0,36)	0,0913	0,6046
**OLP.169**	858	9	4,48 (0,90)	4,76 (0,40)	0,0722	0,2389	630	1	4,47 (0,95)	4,63 (0)	NA	NA	228	8	4,53 (0,74)	4,78 (0,42)	0,1562	0,6863
**OLP.174**	842	25	4,48 (0,90)	4,71 (0,64)	0,0944	0,2603	620	11	4,46 (0,95)	4,83 (0,82)	0,1645	0,3483	222	14	4,54 (0,74)	4,61 (0,45)	0,6010	0,9047
**OLP.176**	850	17	4,48 (0,90)	4,74 (0,48)	0,0455	0,2101	628	3	4,46 (0,95)	5,02 (0,73)	0,3211	0,4386	222	14	4,53 (0,74)	4,68 (0,43)	0,2466	0,7557
**OLP.178**	843	24	4,47 (0,89)	5,09 (0,82)	0,0012	0,0333	615	16	4,45 (0,94)	5,21 (0,89)	0,0037	0,0475	228	8	4,53 (0,73)	4,86 (0,66)	0,2091	0,7487
**OLP.179**	861	6	4,49 (0,90)	4,59 (0,52)	0,6389	0,5354	629	2	4,47 (0,95)	4,28 (0,04)	0,0017	0,0326	232	4	4,54 (0,73)	4,75 (0,60)	0,5276	0,9047
**OLP.180**	834	33	4,48 (0,90)	4,77 (0,67)	0,0200	0,1305	609	22	4,46 (0,96)	4,77 (0,65)	0,0398	0,1749	225	11	4,53 (0,73)	4,77 (0,74)	0,3128	0,8429
**OLP.181**	844	23	4,49 (0,89)	4,35 (0,97)	0,4914	0,4968	620	11	4,48 (0,95)	3,76 (1,00)	0,0396	0,1749	224	12	4,52 (0,74)	4,89 (0,54)	0,0464	0,5196
**OLP.182**	839	28	4,48 (0,90)	4,67 (0,55)	0,0984	0,2684	623	8	4,47 (0,95)	4,49 (0,65)	0,9112	0,6538	216	20	4,53 (0,75)	4,73 (0,50)	0,1019	0,6068
**OLP.183**	826	41	4,48 (0,91)	4,67 (0,63)	0,0765	0,2485	613	18	4,45 (0,96)	4,90 (0,60)	0,0066	0,0597	213	23	4,55 (0,74)	4,48 (0,60)	0,6252	0,9047
**OLP.187**	780	87	4,47 (0,91)	4,65 (0,75)	0,0357	0,1885	571	60	4,44 (0,97)	4,67 (0,77)	0,0397	0,1749	209	27	4,53 (0,73)	4,62 (0,72)	0,5732	0,9047
**OLP.188**	853	14	4,48 (0,90)	5,02 (0,61)	0,0058	0,0631	623	8	4,46 (0,95)	5,10 (0,77)	0,0514	0,1999	230	6	4,53 (0,73)	4,92 (0,37)	0,0477	0,5196
**OLP.190**	764	103	4,46 (0,90)	4,69 (0,81)	0,0101	0,0932	566	65	4,43 (0,95)	4,76 (0,88)	0,0060	0,0597	198	38	4,54 (0,74)	4,56 (0,66)	0,8943	0,9459
**OLP.191**	849	18	4,48 (0,90)	4,73 (0,52)	0,0623	0,2265	620	11	4,46 (0,95)	4,89 (0,60)	0,0411	0,1749	229	7	4,54 (0,74)	4,49 (0,25)	0,6233	0,9047
**OLP.194**	839	28	4,49 (0,90)	4,28 (0,74)	0,1476	0,3215	620	11	4,47 (0,95)	3,99 (0,85)	0,0911	0,2649	219	17	4,55 (0,74)	4,47 (0,61)	0,6131	0,9047
**OLP.195**	860	7	4,49 (0,90)	4,23 (0,84)	0,4523	0,4902	629	2	4,46 (0,95)	5,35 (0,36)	0,1719	0,3517	231	5	4,56 (0,73)	3,79 (0,40)	0,0108	0,2758
**OLP.199**	847	20	4,49 (0,90)	4,16 (0,72)	0,0565	0,2260	623	8	4,48 (0,95)	3,71 (0,80)	0,0290	0,1538	224	12	4,55 (0,74)	4,47 (0,50)	0,6025	0,9047
**OLP.203**	832	35	4,50 (0,89)	4,22 (0,98)	0,1079	0,2728	598	33	4,48 (0,95)	4,19 (1,00)	0,1102	0,3030	234	2	4,54 (0,73)	4,74 (0,06)	0,0189	0,3324
**OLP.206**	860	7	4,49 (0,90)	4,50 (0,42)	0,9216	0,6132	629	2	4,47 (0,95)	4,74 (0,00)	0,0000	0,0000	231	5	4,55 (0,73)	4,41 (0,47)	0,5585	0,9047
**OLP.212**	821	46	4,48 (0,90)	4,68 (0,71)	0,0664	0,2323	603	28	4,45 (0,95)	4,78 (0,82)	0,0512	0,1999	218	18	4,54 (0,75)	4,54 (0,50)	0,9655	0,9508
**OLP.216**	796	71	4,51 (0,89)	4,22 (0,94)	0,0135	0,1099	581	50	4,50 (0,94)	4,08 (1,00)	0,0065	0,0597	215	21	4,54 (0,74)	4,54 (0,69)	0,9774	0,9508
**OLP.220**	849	18	4,50 (0,89)	4,01 (0,90)	0,0348	0,1880	617	14	4,48 (0,95)	3,99 (1,02)	0,1012	0,2865	232	4	4,55 (0,73)	4,05 (0,35)	0,0581	0,5196
**OLP.224**	726	141	4,46 (0,90)	4,63 (0,88)	0,0427	0,2088	506	125	4,42 (0,96)	4,66 (0,88)	0,0074	0,0646	220	16	4,56 (0,72)	4,36 (0,89)	0,3965	0,8771
**OLP.226**	845	22	4,50 (0,90)	4,13 (0,87)	0,0609	0,2260	617	14	4,48 (0,95)	4,01 (1,00)	0,1045	0,2920	228	8	4,55 (0,73)	4,33 (0,60)	0,3376	0,8504
**OLP.228**	848	19	4,48 (0,90)	4,93 (0,77)	0,0198	0,1305	618	13	4,46 (0,95)	4,98 (0,82)	0,0413	0,1749	230	6	4,54 (0,73)	4,84 (0,73)	0,3533	0,8613
**OLP.236**	843	24	4,48 (0,89)	4,85 (0,91)	0,0560	0,2260	615	16	4,46 (0,95)	4,85 (1,03)	0,1484	0,3483	228	8	4,53 (0,73)	4,86 (0,67)	0,2129	0,7487
**OLP.237**	859	8	4,48 (0,90)	4,80 (0,77)	0,2853	0,4141	630	1	4,47 (0,95)	3,18 (0)	NA	NA	229	7	4,53 (0,73)	5,03 (0,43)	0,0212	0,3545
**OLP.238**	857	10	4,48 (0,90)	5,09 (0,73)	0,0281	0,1625	622	9	4,46 (0,95)	5,13 (0,76)	0,0305	0,1544	235	1	4,54 (0,73)	4,72 (0)	NA	NA
**OLP.239**	856	11	4,48 (0,90)	4,99 (0,48)	0,0053	0,0631	628	3	4,46 (0,95)	5,55 (0,05)	0,0000	0,0000	228	8	4,53 (0,74)	4,79 (0,39)	0,1164	0,6068
**OLP.240**	860	7	4,48 (0,90)	5,00 (0,27)	0,0015	0,0340	630	1	4,47 (0,95)	4,74 (0)	NA	NA	230	6	4,53 (0,73)	5,05 (0,26)	0,0028	0,0935
**OLP.243**	852	15	4,49 (0,90)	4,17 (0,78)	0,1371	0,3105	628	3	4,47 (0,95)	3,36 (0,24)	0,0103	0,0737	224	12	4,55 (0,73)	4,38 (0,73)	0,4354	0,9016
**OLP.250**	835	32	4,48 (0,90)	4,67 (0,73)	0,1526	0,3215	620	11	4,46 (0,95)	5,05 (0,91)	0,0564	0,2083	215	21	4,55 (0,75)	4,48 (0,55)	0,5957	0,9047
**OLP.253**	815	52	4,47 (0,89)	4,75 (0,91)	0,0382	0,1935	590	41	4,44 (0,95)	4,87 (0,94)	0,0066	0,0597	225	11	4,56 (0,73)	4,29 (0,64)	0,2034	0,7487
**OLP.259**	829	38	4,48 (0,91)	4,66 (0,58)	0,0791	0,2485	612	19	4,46 (0,96)	4,82 (0,47)	0,0037	0,0475	217	19	4,55 (0,74)	4,49 (0,65)	0,7229	0,9181
**OLP.260**	859	8	4,48 (0,90)	5,06 (0,68)	0,0457	0,2101	627	4	4,46 (0,95)	5,20 (0,77)	0,1527	0,3483	232	4	4,54 (0,73)	4,93 (0,65)	0,3153	0,8429
**OLP.264**	846	21	4,49 (0,90)	4,26 (0,78)	0,1852	0,3536	622	9	4,47 (0,95)	3,88 (0,54)	0,0112	0,0763	224	12	4,54 (0,73)	4,54 (0,83)	0,9853	0,9508
**OLP.265**	768	99	4,46 (0,90)	4,69 (0,86)	0,0169	0,1196	541	90	4,43 (0,96)	4,69 (0,89)	0,0101	0,0737	227	9	4,54 (0,74)	4,60 (0,62)	0,8009	0,9459
**OLP.271**	850	17	4,49 (0,90)	4,33 (0,72)	0,3965	0,4637	627	4	4,46 (0,95)	4,90 (0,62)	0,2510	0,4075	223	13	4,57 (0,73)	4,16 (0,68)	0,0575	0,5196
**OLP.272**	862	5	4,49 (0,90)	4,72 (0,88)	0,5801	0,5152	628	3	4,46 (0,95)	5,30 (0,41)	0,0689	0,2383	234	2	4,55 (0,73)	3,86 (0,54)	0,3225	0,8429
**OLP.277**	861	6	4,49 (0,90)	4,78 (0,59)	0,2812	0,4141	627	4	4,46 (0,95)	4,98 (0,39)	0,0738	0,2448	234	2	4,54 (0,73)	4,36 (0,87)	0,8180	0,9459

**OLP.402**	862	5	4,49 (0,90)	4,74 (0,15)	0,0146	0,1106	630	1	4,47 (0,95)	4,73 (0)	NA	NA	232	4	4,54 (0,74)	4,75 (0,18)	0,0912	0,6046
**OLP.405**	860	7	4,49 (0,90)	4,06 (0,79)	0,1975	0,3659	630	1	4,47 (0,95)	5,04 (0)	NA	NA	230	6	4,56 (0,72)	3,89 (0,72)	0,0740	0,5693
**OLP.406**	857	10	4,49 (0,90)	4,23 (0,71)	0,2828	0,4141	628	3	4,46 (0,95)	4,88 (0,25)	0,0911	0,2649	229	7	4,56 (0,73)	3,95 (0,66)	0,0506	0,5196
**OLP.413**	849	18	4,48 (0,90)	4,74 (0,90)	0,2545	0,4055	617	14	4,46 (0,95)	4,92 (0,84)	0,0595	0,2163	232	4	4,55 (0,73)	4,09 (0,94)	0,3958	0,8771
**OLP.417**	863	4	4,49 (0,89)	3,53 (0,90)	0,1215	0,2928	628	3	4,47 (0,95)	3,10 (0,32)	0,0150	0,0963	235	1	4,54 (0,73)	4,82 (0)	NA	NA
**OLP.421**	859	8	4,48 (0,90)	5,05 (0,85)	0,1016	0,2700	623	8	4,46 (0,95)	5,05 (0,85)	0,0910	0,2649	236	0	4,54 (0,73)	0 (0)	NA	NA
**OLP.279**	841	26	4,48 (0,90)	4,81 (0,68)	0,0237	0,1441	627	4	4,47 (0,95)	4,62 (0,40)	0,4945	0,5288	214	22	4,51 (0,73)	4,84 (0,72)	0,0539	0,5196
**OLP.280**	845	22	4,48 (0,90)	4,70 (0,55)	0,0789	0,2485	622	9	4,46 (0,95)	4,88 (0,56)	0,0542	0,2037	223	13	4,54 (0,74)	4,58 (0,52)	0,8196	0,9459
**OLP.281**	729	138	4,50 (0,88)	4,41 (0,97)	0,2727	0,4061	507	124	4,49 (0,94)	4,36 (0,99)	0,1672	0,3483	222	14	4,52 (0,73)	4,83 (0,62)	0,0961	0,6046
**OLP.283**	790	77	4,46 (0,91)	4,73 (0,71)	0,0034	0,0525	579	52	4,44 (0,96)	4,75 (0,81)	0,0105	0,0737	211	25	4,53 (0,76)	4,67 (0,45)	0,1930	0,7487
**OLP.284**	806	61	4,47 (0,91)	4,74 (0,72)	0,0060	0,0631	585	46	4,44 (0,96)	4,80 (0,78)	0,0053	0,0576	221	15	4,54 (0,75)	4,59 (0,47)	0,7153	0,9181
**OLP.286**	852	15	4,49 (0,89)	4,54 (1,07)	0,8465	0,5915	623	8	4,47 (0,95)	4,17 (1,29)	0,5367	0,5481	229	7	4,53 (0,73)	4,96 (0,58)	0,0976	0,6046
**OLP.287**	850	17	4,49 (0,90)	4,56 (0,70)	0,6694	0,5445	620	11	4,47 (0,95)	4,35 (0,73)	0,5926	0,5553	230	6	4,53 (0,73)	4,96 (0,47)	0,0767	0,5693
**OLP.288**	858	9	4,48 (0,90)	5,00 (0,70)	0,0572	0,2260	624	7	4,46 (0,95)	4,90 (0,72)	0,1599	0,3483	234	2	4,54 (0,73)	5,37 (0,71)	0,3437	0,8504
**OLP.115**	837	30	4,48 (0,90)	4,73 (0,80)	0,1010	0,2700	613	18	4,46 (0,95)	4,62 (0,95)	0,4804	0,5260	224	12	4,52 (0,74)	4,89 (0,52)	0,0359	0,5196
**OLP.116**	835	32	4,47 (0,90)	4,94 (0,79)	0,0022	0,0460	612	19	4,45 (0,95)	5,07 (0,72)	0,0016	0,0326	223	13	4,53 (0,72)	4,75 (0,86)	0,3788	0,8771
**OLP.117**	859	8	4,49 (0,89)	4,65 (1,09)	0,6756	0,5462	625	6	4,47 (0,95)	4,54 (1,27)	0,8848	0,6538	234	2	4,54 (0,73)	4,98 (0,01)	0,0000	0,0000
**OLP.100**	828	39	4,47 (0,89)	4,75 (0,97)	0,0938	0,2603	595	36	4,45 (0,94)	4,77 (1,00)	0,0676	0,2376	233	3	4,54 (0,73)	4,46 (0,43)	0,7784	0,9385
**OLP.102**	837	30	4,48 (0,90)	4,74 (0,77)	0,0810	0,2513	609	22	4,45 (0,95)	4,88 (0,85)	0,0317	0,1569	228	8	4,55 (0,74)	4,36 (0,23)	0,0604	0,5196
**OLP.289**	861	6	4,49 (0,90)	4,54 (0,79)	0,8775	0,6050	627	4	4,47 (0,95)	4,60 (1,01)	0,8037	0,6317	234	2	4,54 (0,73)	4,41 (0,04)	0,0394	0,5196
**OLP.292**	859	8	4,48 (0,90)	4,93 (0,55)	0,0563	0,2260	629	2	4,46 (0,95)	5,09 (1,11)	0,5732	0,5553	230	6	4,53 (0,74)	4,87 (0,40)	0,0937	0,6046
**OLP.300**	863	4	4,48 (0,90)	5,03 (0,40)	0,0693	0,2387	629	2	4,46 (0,95)	5,29 (0,44)	0,2220	0,3834	234	2	4,54 (0,73)	4,76 (0,03)	0,0001	0,0118
**OLP.301**	860	7	4,48 (0,90)	4,92 (0,53)	0,0721	0,2389	629	2	4,47 (0,95)	4,70 (0,24)	0,3922	0,4857	231	5	4,53 (0,73)	5,02 (0,62)	0,1547	0,6863
**OLP.306**	865	2	4,49 (0,90)	4,80 (0,10)	0,0937	0,2603	630	1	4,47 (0,95)	4,73 (0)	NA	NA	235	1	4,54 (0,73)	4,87 (0)	NA	NA
**OLP.307**	864	3	4,48 (0,90)	5,15 (0,17)	0,0151	0,1106	631	0	4,47 (0,95)	0 (0)	NA	NA	233	3	4,54 (0,73)	5,15 (0,17)	0,0115	0,2758
**OLP.311**	860	7	4,48 (0,90)	5,24 (0,47)	0,0048	0,0631	629	2	4,46 (0,95)	5,09 (0,36)	0,2437	0,4024	231	5	4,53 (0,73)	5,30 (0,53)	0,0291	0,4440
**OLP.312**	861	6	4,48 (0,90)	5,22 (0,54)	0,0197	0,1305	628	3	4,46 (0,95)	5,63 (0,43)	0,0385	0,1749	233	3	4,54 (0,73)	4,80 (0,13)	0,0411	0,5196
**OLP.314**	840	27	4,48 (0,90)	4,85 (0,58)	0,0029	0,0509	627	4	4,47 (0,95)	4,55 (0,96)	0,8671	0,6501	213	23	4,50 (0,74)	4,90 (0,50)	0,0017	0,0720
**OLP.315**	849	18	4,48 (0,90)	4,86 (0,52)	0,0068	0,0676	631	0	4,47 (0,95)	0 (0)	NA	NA	218	18	4,52 (0,74)	4,86 (0,52)	0,0156	0,3246
**OLP.319**	863	4	4,49 (0,90)	4,91 (0,35)	0,0937	0,2603	630	1	4,47 (0,95)	4,81 (0)	NA	NA	233	3	4,54 (0,73)	4,94 (0,42)	0,2375	0,7487
**OLP.321**	856	11	4,48 (0,90)	4,66 (0,43)	0,2227	0,3896	627	4	4,46 (0,95)	5,07 (0,31)	0,0245	0,1341	229	7	4,55 (0,74)	4,42 (0,29)	0,3169	0,8429
**OLP.331**	865	2	4,49 (0,90)	4,39 (0,04)	0,0610	0,2260	631	0	4,47 (0,95)	0 (0)	NA	NA	234	2	4,54 (0,73)	4,39 (0,04)	0,0124	0,2758
**OLP.332**	864	3	4,49 (0,90)	4,44 (0,62)	0,9049	0,6103	630	1	4,47 (0,95)	3,73 (0)	NA	NA	234	2	4,54 (0,73)	4,79 (0,10)	0,0936	0,6046
**OLP.335**	859	8	4,48 (0,90)	4,85 (0,50)	0,0783	0,2485	626	5	4,46 (0,95)	4,95 (0,61)	0,1495	0,3483	233	3	4,54 (0,73)	4,68 (0,25)	0,4277	0,9016
**OLP.343**	864	3	4,49 (0,90)	4,89 (0,22)	0,0822	0,2517	630	1	4,47 (0,95)	4,92 (0)	NA	NA	234	2	4,54 (0,73)	4,88 (0,32)	0,3651	0,8771
**OLP.355**	862	5	4,49 (0,90)	4,50 (1,03)	0,9736	0,6295	629	2	4,47 (0,95)	3,53 (0,99)	0,4057	0,4883	233	3	4,53 (0,73)	5,16 (0,16)	0,0084	0,2538
**OLP.356**	862	5	4,48 (0,90)	5,07 (0,45)	0,0438	0,2090	628	3	4,46 (0,95)	4,95 (0,60)	0,2928	0,4342	234	2	4,54 (0,73)	5,25 (0,05)	0,0000	0,0016
**OLP.360**	859	8	4,48 (0,90)	5,16 (0,73)	0,0332	0,1834	624	7	4,46 (0,95)	5,21 (0,77)	0,0410	0,1749	235	1	4,54 (0,73)	4,80 (0)	NA	NA
**OLP.362**	848	19	4,48 (0,89)	4,81 (0,90)	0,1328	0,3105	614	17	4,45 (0,95)	4,91 (0,89)	0,0506	0,1999	234	2	4,55 (0,73)	3,92 (0,50)	0,3178	0,8429
**OLP.365**	828	39	4,46 (0,89)	5,03 (0,91)	0,0005	0,0165	593	38	4,43 (0,94)	5,05 (0,91)	0,0002	0,0075	235	1	4,54 (0,73)	4,19 (0)	NA	NA
**OLP.371**	842	25	4,48 (0,90)	4,66 (0,79)	0,2718	0,4061	613	18	4,46 (0,95)	4,81 (0,81)	0,0836	0,2602	229	7	4,55 (0,73)	4,28 (0,67)	0,3385	0,8504
**OLP.378**	863	4	4,49 (0,90)	4,87 (0,23)	0,0429	0,2088	631	0	4,47 (0,95)	0 (0)	NA	NA	232	4	4,54 (0,73)	4,87 (0,23)	0,0586	0,5196

**OLP.381**	863	4	4,48 (0,90)	4,97 (0,31)	0,0508	0,2249	629	2	4,46 (0,95)	5,19 (0,22)	0,1154	0,3030	234	2	4,54 (0,73)	4,75 (0,22)	0,3997	0,8771
**OLP.393**	859	8	4,48 (0,89)	5,24 (1,15)	0,1034	0,2701	626	5	4,45 (0,94)	5,99 (0,57)	0,0034	0,0475	233	3	4,55 (0,73)	3,99 (0,42)	0,1433	0,6839
**OLP.396**	851	16	4,49 (0,90)	4,57 (0,77)	0,6569	0,5416	623	8	4,47 (0,95)	4,24 (0,92)	0,4994	0,5288	228	8	4,53 (0,74)	4,91 (0,39)	0,0293	0,4440
**OLP.397**	843	24	4,48 (0,90)	4,75 (0,84)	0,1376	0,3105	620	11	4,46 (0,95)	5,01 (0,93)	0,0791	0,2500	223	13	4,54 (0,73)	4,52 (0,70)	0,9170	0,9508
**OLP.398**	859	8	4,49 (0,90)	4,01 (0,70)	0,0926	0,2603	629	2	4,47 (0,95)	3,24 (0,91)	0,3049	0,4345	230	6	4,55 (0,73)	4,26 (0,47)	0,1945	0,7487
**OLP.399**	864	3	4,48 (0,90)	5,52 (0,24)	0,0137	0,1099	630	1	4,46 (0,95)	5,51 (0)	NA	NA	234	2	4,53 (0,73)	5,53 (0,34)	0,1385	0,6705
**OLP.401**	841	26	4,48 (0,90)	4,77 (0,61)	0,0238	0,1441	631	0	4,47 (0,95)	0 (0)	NA	NA	210	26	4,51 (0,74)	4,77 (0,61)	0,0540	0,5196
**OLP.67**	861	6	4,48 (0,90)	4,88 (0,53)	0,1288	0,3044	629	2	4,47 (0,95)	4,77 (1,11)	0,7651	0,6179	232	4	4,54 (0,73)	4,93 (0,20)	0,0184	0,3324
**OLP.71**	859	8	4,48 (0,90)	4,95 (0,62)	0,0707	0,2389	624	7	4,46 (0,95)	5,12 (0,42)	0,0052	0,0576	235	1	4,55 (0,73)	3,76 (0)	NA	NA
**OLP.76**	574	293	4,45 (0,90)	4,55 (0,89)	0,1254	0,2993	414	217	4,46 (0,96)	4,48 (0,94)	0,8664	0,6501	160	76	4,43 (0,73)	4,77 (0,68)	0,0007	0,0370
**OLP.79**	792	75	4,47 (0,90)	4,67 (0,84)	0,0583	0,2260	592	39	4,45 (0,96)	4,70 (0,80)	0,0664	0,2374	200	36	4,53 (0,70)	4,62 (0,88)	0,5398	0,9047
**OLP.83**	763	104	4,47 (0,91)	4,63 (0,81)	0,0550	0,2260	577	54	4,45 (0,95)	4,60 (0,94)	0,2689	0,4208	186	50	4,51 (0,75)	4,67 (0,65)	0,1457	0,6855
**OLP.84**	605	262	4,45 (0,92)	4,58 (0,82)	0,0301	0,1700	432	199	4,44 (0,98)	4,53 (0,89)	0,2309	0,3933	173	63	4,47 (0,78)	4,75 (0,52)	0,0020	0,0724
**OLP.85**	695	172	4,45 (0,91)	4,62 (0,84)	0,0244	0,1441	512	119	4,45 (0,95)	4,54 (0,95)	0,3211	0,4386	183	53	4,47 (0,77)	4,79 (0,51)	0,0006	0,0370
**OLP.89**	859	8	4,49 (0,90)	4,46 (0,83)	0,9207	0,6132	629	2	4,47 (0,95)	4,69 (0,07)	0,0433	0,1783	230	6	4,55 (0,72)	4,38 (0,97)	0,6893	0,9084
